# Service Users’ Perceptions of an Outreach Wellbeing Service: A Social Enterprise for Promoting Mental Health

**DOI:** 10.1007/s10597-016-0079-2

**Published:** 2017-01-17

**Authors:** Sandra Elaine Hartley

**Affiliations:** Department of Health Professions, Manchester Metropolitan University, Brooks Building, Birley Campus, 53 Bonsall Street, Manchester, M15 6GX UK

**Keywords:** Self-management, Collaborative partnerships, Wellbeing, Depression, Social enterprise

## Abstract

Inadequate provision and limited access to mental healthcare has been highlighted with the need to offer more contemporary ways to provide clinically effective interventions. This study aimed to present an insight into service users’ perceptions of an outreach Wellbeing Service (WBS), providing psychological therapy in social settings. Descriptive and thematic analysis was undertaken of 50 returned surveys. Comparison of initial and final mental health measures demonstrated a significant improvement in all outcomes with 96% of participants reporting being helped by attending. Participants were assisted to rebuild social connections in a safe and supportive environment and were facilitated to become more self-determining as their resourcefulness to self-manage was cultivated. Situated within different settings within the community, the WBS offers a workable example of a novel approach to supporting and promoting citizens to become more resilient and lead a more fulfilling and independent life in the community.

## Introduction

Mental ill health is one of the major causes of disability accounting for 13% of the global disease burden (World Health Organisation (WHO) 2008; Bloom et al. 2011). The total world expenditure due to lost productivity because of mental ill health has been predicted to reach £11.3 trillion in the next 20 years (Whiteford et al. 2013). In England alone, the annual health and social care budgets for mental ill health, is estimated to be 21.3 billion (Centre for mental health 2010) with the prediction that the cost of mental health interventions will double by 2026 (McCrone et al. 2008). A quarter of individuals will experience mental ill health at some point in their lifetime, with depression and anxiety being the most common (McManus et al. 2009; Whiteford et al. 2013). Depression and anxiety have a detrimental effect on quality of life and social functioning (McCrone et al. 2008; DH 2011) and can lead to early mortality (Friedli 2009; WHO 2010). Depression alone is projected to be the highest cause of disease burden by 2030 (WHO 2008) yet, only one in four individuals with depression and anxiety receive treatment (NHS England (NHSE) 2014; The Mental Health Policy Group (MHPG) 2014). This is predominately due to the inadequate provision of mental healthcare and limited access to evidence based interventions, specifically psychological therapies, with many individuals being deterred from seeking help (Gask et al. 2012; DH 2014). In an endeavour to provide more equitable and timely access to clinically effective interventions, a UK Government initiative, ‘Improving Access to Psychological Therapies’ (IAPT) has been implemented. This scheme offers sponsorship for the training of additional psychological therapists, in a drive to increase the workforce that have the skills to offer clinically effective psychological interventions (Centre for Workforce intelligence 2013; NHSE 2015; Wolitzky-Taylor et al. 2015). Further, maximum waiting time targets have been set, with the expectation that 75% of people will have accessed relevant treatment within 6 weeks of referral and 95% by 18 weeks (The Royal College of Psychiatrists 2014; NHSE 2015). However, this comes at a time of public sector austerity where health and social care budgets are diminishing (Foley 2013; MHPG 2014). The prospect of delivering more for less is untenable, without finding more innovative ways to provide these essential services (Hanlon et al. 2011; Ham et al. 2012).

Social enterprises (SEs), who seek to improve the welfare of its citizens through local collaboration and social change (Bull 2006; Park and Wilding 2014), are being seen as playing significant roles in promoting public health (Roy et al. 2014). Often rooted within the community and having close ties with its people, they have the potential to offer more pioneering approaches to healthcare (Peattie and Morley 2008; Roy et al. 2014) and could bridge the gap between health care services and their citizens (Addicott 2011; WHO 2013). A not for profit social enterprise, located in a city district of England, has been established for people with mental ill health. As part of a personalised mental health and wellbeing, recovery programme it provides solution focused interventions and facilitates self-management. One of its initiatives has been the establishment of an outreach service for local people with psychological distress. Three wellbeing workers (WBWs), who have been IAPT trained in psychological therapy, offer this facility as a first line intervention for depression and anxiety disorders. The WBWs are all non-specialist who, prior to their training, had no previous qualifications in mental health but had experience of working with people with mental ill health or an interest in this area. Although a service is provided in the local GP practice, the main tenet is the provision of psychotherapy in social settings with the WBWs being situated within different community locations. The WBWs may also; direct individuals to their local partner organisations, depending upon the person’s own personal needs, for example, exercise and leisure activities, complementary therapies, and other regional social services. Although people can self-refer to the WBWs, many attend following recommendation from their GP and other community services. This study aims to offer an insight into the service provision provided by the WBWs and to elicit service users’ views, to inform and help shape future service delivery.

## Objectives

To describe patterns of attendance at the wellbeing service (WBS).

To explore service users perceptions of the value of the service provided by the WBWs for improving mental wellbeing.

To measure the effectiveness of the WBS in improving mental wellbeing.

## Methods

A survey design and retrospective data collection was undertaken. Following feedback from a pilot study of nine adults, an additional question requesting waiting time from referral to attending first appointment was added, resulting in a finalised 16-item questionnaire. Closed questions mainly concerning aspects of attendance, skills of the WBWs and the service provision were included. Additionally, open questions were incorporated to explore further, participants’ views of service provision to gain more depth of understanding. One hundred and seventy-two questionnaires, with a covering letter detailing the study and requesting consent both to take part in the study and to access service users’ records, were mailed out to individuals who had attended the WBS over the previous 18 months. To promote the survey further, posters with study details and contact information were placed in local community facilities. Due to only 12 returned postal questionnaires, other recruitment methods were employed including an invite and an electronic link to the survey sent to; personal e-mail addresses, mobile telephones, placed on the WBS facebook page and tweeted to the organisations twitter account. Including the pilot study, this resulted in 50 respondents between the 5th of February and the 16th of April 2015.

## Data Analysis

All data from returned questionnaires were inputted in to SPSS version 21. This included information that had been extracted from the participants’ documentation namely, demographics, interventions, and the first and last outcome measure scores from the Warwick-Edinburgh Mental Well-being Scale (WEMWBS), the generalised anxiety disorder scale (GAD −7) and the patient health questionnaire (PHQ-9). One participant had attended for a one off consultation and was therefore, not included in the calculations. Two participants had final data for the WEMWBS only and were therefore, excluded from GAD-7 and the PHQ-9 statistical tests. Test of normality for all outcome measures was carried out using the Shapiro–Wilk test. This revealed that the data for the WEWBS (n = 49) was normally distributed (p > 0.05) therefore; the paired t-test was used to compare initial and final scores. However, the data for the GAD- 7 (n = 47) and PHQ-9 (n = 47) were not normally distributed (both p < 0.05) therefore, the Wilcoxon signed Rank Test was carried out to compare initial and final test scores for both these measures (see Table 1 for scores of severity of depression (PHQ-9) and anxiety (GAD-7) at initial attendance and final assessment). Descriptive analysis of all closed questions was also undertaken.

**Table 1:** Depression and anxiety scores at initial and final assessment

	Initial assessment n (%)	Final assessment n (%)
Depression severity (PHQ-9 scores)		
None/Minimal (0–4)	6 (12)	15 (32)
Mild (5–9)	8 (16)	16 (34)
Moderate (10–14)	12 (25)	8 (17)
Moderately severe (15–19)	13 (27)	7 (15)
Severe (20–27)	10 (20)	1 (2)
Anxiety severity (GAD-7 scores)		
None/Minimal (0–4)	2 (4)	11 (23)
Mild (5–9)	9 (18)	23 (49)
Moderate (10–14)	20 (41)	9 (19)
Severe (15–21)	18 (37)	4 (9)
Total number of participants	49	47

A thematic analysis of the open questions was carried out following the process highlighted by Braun and Clarke (2006). The researcher read iteratively the written text to gain more understanding of the data collected, before coding the data. Codes were derived based on important concepts that were identified and words or phrases that were reoccurring within the data. Codes that were deemed as similar were then collated together under a common heading to form a subtheme. Subthemes that were related were then grouped together into main themes and relabelled based on what appeared to epitomise the essence of what the data was saying (see Table 2). The analysis was a fluid process, with the researcher going backwards and forwards between the themes and representing data to ensure authenticity and that no theme remained redundant. Memos written during this time also, aided the analytical process (Braun and Clarke 2013). To enhance credibility of the findings and challenge researcher assumptions, debriefing with a colleague took place and a peer review from a supervisory team was regularly undertaken.

**Table 2:** Themes identified from participants perceptions of the value of the wellbeing workers and the service provision

Themes	Subthemes	Codes
Supportive environment	Service provision	personalised care flexible service place for solace someone to talk to
	Worker characteristics	empathetic
		non-judgemental
		friendly
		dependable
Self-determining	Knowledge exchange	strategies for coping
		advice and education
		techniques to assist self-management
	Sense of hope	hope for the future
		assistance to get well
Rebuilding connections	Develop positive relationship with self	better self-understanding
		validation of condition
		improved self-assurance
	Develop positive relationships with others	new perspective of circumstances
		more considerate of others
		greater awareness of situation
		improved communication
	Greater affinity to others	more sociable
		reconnect with friends
Service enhancement	Appointment times	more out of hours
		reduce waiting times
	Service provision	increase staffing
		more funding
		advertise more

## Results

Fifty individuals (39 females) completed the questionnaire with mean ages, males 46.9 (SD 13.31; range 22–66 years); females 43.6 (SD 15.4; range 21–74 years) (see Table 3 for further demographic information).

**Table 3:** Demographics of service users who participated in the survey

	n	%
Gender		
Male	11	22
Female	39	78
Age in years		
Mean (SD)	44 (14.9)	
Range	Range 21–74	
Ethnicity		
White British	44	88
White European	2	4
Mixed-Black and Chinese	1	2
Asian other—Sinhalese	1	2
Asian other—East African Asian	1	2
Black or Black British African	1	2
Employment		
Full-time	16	32
Part-time	12	24
Unemployed	11	22
Economically inactive	5	10
Retired	4	8
In education	1	2
Career sabbatical	1	2

Total participants n = 50

Forty-six (92%) reported attending the service due to anxiety and depression or difficulty coping, two (4%) due to an eating disorder, one (2%) due to a court order and one (2%) was a non-responder. Eighteen (36%) were experiencing pain when first attending the service with five (28%) reporting that they were helped to a great extent to live with the pain, 61% being somewhat helped, one (5.6%) helped very little and one (5.6%) not helped at all.

The majority of participants were signposted to the WBWs by their GP’s. Of the 31 participants who reported waiting time for appointment, 64% (20) waited 6 weeks or less, and everyone were seen by 4 months. The mean number of times attended was 7.84 (SD 7.08; range 1–34). Most of the participants accessed the WBWs at least once a fortnight and 44 (88%) reported this was an appropriate amount of time. No participant reported that frequency of attendance was too much (see Table 3). Although most participants received a combination of interventions, most commonly used was psychological therapy (Table 4).

**Table 4:** Type of interventions received by participant

Type of interventions	Psychological therapy^a^ n (%)	Problem solving^b^ n (%)	Proactivity^c^ n (%)	Employment^d^ n (%)
Number of participants n = 50	50 (100)	39 (78)	32 (64)	20 (40)

^a^Includes CBT, active listening, relaxation, classic thought challenging, mindfulness

^b^ Includes advice, education, sign posting to other activities or services in the community

^c^ Includes behavioural activation, engaging in hobbies/interests

^d^Includes job club, careers advice, volunteering opportunities

Forty-eight (96%) of participants were satisfied with the therapy they received from the WBWs. Forty-four (88%) agreed that the WBWs had the appropriate skills to provide psychological support, 46 (92%) felt they had a good understanding of their circumstances, 47 (94%) agreed that they were involved in the decision making process with their WBW, 46 (92%) felt the WBWs were focused on their individual needs and 46 (92%) felt the WBWs were empathetic. Thirty-three (66%) reported that accessing the WBS had helped them to a great extent, 13 (26%) felt that it had somewhat helped them, two (4%) felt that they had been helped very little, one (2%) felt that they had not been helped at all and one (2%) was a non-responder (see Table 5). Only one participant strongly disagreed that the WBWs had the skills to provide the appropriate psychological support. They were also, very unsatisfied with the service provision and felt that attending the service had made their situation worse.

**Table 5:** Participants perceptions of the wellbeing workers and their satisfaction with the service provision

About the wellbeing workers	Strongly agree n (%)	Agree n (%)	Neutral n (%)	Disagree n (%)	Strongly disagree n (%)	Non responder n (%)
Had the skills to provide appropriate psychological support	35 (70)	9 (18)	3 (6)	1 (2)	1 (2)	1 (2)
Had a good understanding of my circumstances	36 (72)	10 (20)	1 (2)	1 (2)	1 (2)	1 (2)
Included me in making decisions about my therapy	37 (74)	10 (20)	1 (2)	1 (2)	0 (0)	1 (2)
Was focused on my own individual needs	38 (76)	8 (16)	2 (4)	1 (2)	0 (0)	1 (2)
Was empathetic	42 (84)	4 (8)	2 (4)	1 (2)	0 (0)	1 (2)

About service provision	Very satisfied n (%)	Satisfied n (%)	Neutral n (%)	Unsatisfied n (%)	Very unsatisfied n (%)	Not answered n (%)
Level of satisfaction with the service the well-being workers provided	40 (80)	8 (16)	0 (0)	0 (0)	1 (2)	1 (2)

All tests for outcome measures demonstrated a significant improvement following attendance at the WBS (all p < 0.001).

## Themes

There were four main themes identified. These were supportive environment, self-determining, rebuilding connections and service enhancement. The themes will be presented with the corresponding direct quotes from the participants. All names have been changed to ensure participant anonymity.

### Supportive Environment

Participants reported that they felt comfortable with the WBWs as they found them to be *“warm, friendly and down to earth”*. What seemed to be particularly beneficial for their recovery was that they had someone they could talk to, other than *“family or friends”*, who were dependable and would listen to them in an empathetic and non-judgemental way. It was by providing a supportive environment in this way that appeared to give them the credence to “open up” and communicate their situation:

*Knowing I had*/*have a regular fortnightly appointment with someone who I feel empathises with me and who I have come to trust means a great deal to me. Even the fact of leaving the house and being able to talk to someone openly and frankly without fear of being judged is a real relief. It has*/*is also helping me develop the tools to manage my depression in the future, which I have never had before*.

It seemed that having access to the WBS, provided a lifeline for some, a place to go to gain solace when “not coping” with their situation.

*I think there is very little help out there when I was having difficult*/*suicidal thoughts. I think this service helped to save my life, there should be more centres around. I think not enough is spent on mental health and GP doctors only have 15-minute slots, which isn’t enough to treat the core issues. I was lucky and grateful to receive help in a very dark area of life. One of my friends committed suicide, as he got no help. It really is a vital resource*.

The WBWs were perceived by some participants to offer a flexible service, being accommodating when arranging appointments and providing variability in frequency of attendance, depending on participants’ individual needs. There also, appeared to be the opportunity to re-attend if there was any future deterioration in their condition.

*I never felt rushed during the sessions, and I was also able to access the service for much longer than I expected. Towards the completion, my worker was very flexible in terms of trailing off the sessions -it didn’t stop abruptly, it was when I was ready …*”

“[*I*] *stopped because I had progressed and not needed to go anymore. Told I could access again if wanted*.

However, this was not a universal finding, as there was also the suggestion from one participant that the provision of additional appointments would been more beneficial for some, to support their return to wellbeing.

*There is a limit on the number of sessions, and I didn’t think ‘one size fits all’. Some people will need more sessions to aid their recovery*

### Self-Determining

Many participants reported that the WBWs shared with them, advice about their condition and supported them to become aware of techniques and strategies to aid them to cope with their situation. Providing assistance in this way, and encouraging participants to take part in decision making about their management, facilitated participants to become more self-determining as they developed their ability to manage themselves and their life situation.

………*Specific strategies were also helpful. It was a good blend of theory and practical application. I also felt that Susan had a lot of time for me. There was no sense of being rushed or dealt with’. This made me relax and feel like I was going to be taken seriously. She was also good at reassuring me that the problem could be fixed - again this was reassuring. She gave me a link to a relaxation audio session. Doing this for a couple of weeks has given me another strategy for coping, which I am putting into practice now*.

What seemed significant for participants as they become more involved in their own management is that they started to feel more in control over their situation. There was also the sense of *“hope for the future”* as a *“brighter outlook”* was now seen to be possible, as participants began to believe they could get well again.

*…...I felt as if I had a bit of control of my life I cannot express how important that is*.

*Trust, time and hope. Trust I genuinely believe he is trying to help me and has no other agenda. This is very important to me. Time I don’t feel pressurised at how long it takes to progress and get better, again very important. Hope for the first time in so many years I feel that I will get better and there is a future worth trying for*.

### Rebuilding Connections

Participants began to rebuild connections as they developed relationships that were more positive, both with themselves and with others. Self-acceptance and self-assurance were fostered through validation of their condition from the WBWs and by gaining more insight about their situation.

*Being given a rationale scientific explanation was very helpful- suddenly it wasn’t my own weirdness!* …

*…….. Susan was brilliant with me, dispelled any fears or pre-loaded stigma I had regarding ‘seeing a shrink’, gave me confidence back in a matter of weeks*.

The WBWs helped participants to gain a new perspective of their circumstances, assisting them to deal with their daily circumstances and become more considerate and tolerant of others. In addition, developing the confidence to communicate how they felt with their loved ones helped their families to become more aware of what they were experiencing.

*David my WBW was excellent. He helped me so much. Without his help, I don’t think I would be here today. We talked each time and I began to see things in a different light. He enabled me to cope with certain situations and I grew more confident to handle my life day to day. He was a lifeline…*... .

*My moods and worries would put pressure on my loved ones and take over but my sessions with Susan made it easier for me to open up and discuss my feelings rationally, so they had more of an accurate and calm understanding of what I was going through*.

Becoming more self-assured and responsive to others, supported participants to become more sociable, helping them to develop a greater sense of affinity to other individuals. In some cases, participants contacted old friends whom they had previously shunned due to their condition.

*I feel much more connected with people in general and close relationships with friends. I never realised I had such trouble trusting people after seeing David - it has really ‘opened me up*.

*I have reconnected with 2 close friends who I had stopped contacting as I isolated myself*.

### Service Enhancement

Nevertheless, some improvements to the service were reported. There were requests for more appointments out of the normal hours of delivery specifically, from participants who could not attend due to work commitments. There was also suggestions for more advertising, to improve awareness of the service:

*Yes, I feel there needs to be a service for people who are holding a job down and are so in need of love and support. There needs to be Saturday opening’s and late nights. I came out of work and crashed but some people are unwell and struggling along without help, their families could all be under pressure and this service could help their situation*.

*It should be offered more, should be advertised more put out there and offered instead of being given anti -depressants etc*.

Additionally, what seemed to be a particular concern for some participants was the long waiting time they had before their first appointment and how this had made their situation worse.

*It would be great if an appointment could be given quite quickly to a new client, as waiting, waiting, waiting - increases stress-which is already there in abundance. So speed up the first meeting if possible, which then gives hope and reassurance*.

However, it was clear that people were also, aware that to ensure improvements took place there would need to be more funding available, so that more staff could be employed to help support these changes in the service provision.

*More funding to increase paid workers. Although, I didn’t have to wait long for an appointment, I am aware that others have had a long wait. This investment would pay off as early intervention can reduce long-term costs to MH services and also more importantly benefit the client*/*patient*.

## Discussion

The findings from this study provide an insight into the value of attending an outreach WBS for people with mental ill health. Accessing the WBWs led to a significant improvement in all measures of wellbeing with the majority of participants reporting satisfaction with the service provision and that it helped their situation. Therefore, providing this service may offer a more cost effective intervention by potentially, reducing GP consultations and specialist mental health referrals (Harkness and Bower 2009). Nevertheless, most of the participants were directed to the WBWs by their GP however, people with mental ill health often do not seek medical assistance (McCrone et al. 2008; DH 2014). Therefore, many individuals who may benefit from such a service could be missing out. Finding alternative ways to reach and encourage the attendance of those who are less inclined to do so, provides the opportunity for quicker resolution of symptoms and the prevention of chronic manifestations and the ensuring social and economic consequences (Social Care, Local Government and Care Partnership Directorate (SCLGCPD) 2014). The WBWs by offering facilities in different settings and therefore, being more accessible in their community, could potentially, help to improve awareness of mental ill health and promote the importance of wellbeing to all its citizens (WHO 2013; SCLGCPD 2014). Additionally, providing access to interventions in locations other than medical settings could reduce the stigmatisation often felt by people accessing mental health services and therefore, encourage more people to attend (Clement et al. 2015; Coverdale and Long 2015). Further research to investigate opportunities for mental health promotion and the cost effectiveness of providing this service needs to be undertaken.

Notably, more than a third of the participants were experiencing physical pain when first accessing the WBWs. Depression and anxiety are common features of persistent pain and individuals with a history of psychological distress are at a greater risk of progressing to chronic and incapacitating pain and consequently, perpetuating the mental health condition (Linton and Shaw 2011). Accessing the WBWs helped over a quarter of participants with pain, to live well in spite of their symptoms. This resonates with previous research that has found that psychological interventions help to reduce pain (Scottish Intercollegiate Guidelines Network 2013; Sturgeon 2014). Nevertheless, 72% were still, at the most, somewhat living well with the pain. Considering the strong link between pain and mental ill health (Purdie and Morley 2015), targeting more resources to support the mental health of people experiencing persistent pain could prevent the progression to chronic incapacitating pain and enduring mental ill health thus, enhancing citizen wellbeing. Facilitating the creation of self-help groups in the community, as the peer support and knowledge exchanged between members has been found to be beneficial (Platow et al. 2007), could potentially provide another cost effective way to offer support to people with chronic pain in addition, to reaching a greater number of people (Cruwys et al. 2014). Further research is needed to investigate further, ways to support people with chronic pain to manage their condition in the community.

The WBWs, facilitated participants to become more self-aware and educated about their situation, and assisted them to develop strategies to help them to cope. By supporting individuals to become more resourceful in this way, opportunities were provided for participants to become more self-determining, as participants learnt to draw on their own assets to support their individual needs (Hibbard and Gilburt 2014; Gilburt et al. 2014). Indeed, powerlessness and lack of control over one’s life are main features experienced by people with mental ill health (Crepaz-Keay 2010; McEvoy et al. 2012). Therefore, feeling empowered to self-manage will help to foster wellbeing and a greater life satisfaction (Coote and MacLeod 2012; Greenaway et al. 2015). However, some individuals may not wish or, have the capability to take charge of their own management (NHSE 2013; Hibbard and Gilburt 2014). The WBWs by encouraging involvement in decisions about their management, facilitated individuals to take some responsibility for their situation, which may help to instil confidence in their ability to take further charge (Sterling et al. 2010). Also, the WBWs by instilling hope that there was a better future worth trying for, may offer the encouragement needed for individuals to want to get more involved (Connell et al. 2012; Dalum et al. 2015).

The WBWs provided a supportive environment where they were seen to be empathetic, dependable, non-judgmental and friendly. They were also, perceived by the majority of participants to have a good understanding of their circumstances and were focussed on their individual needs thus, demonstrating an insight into participants’ worlds. Possessing interpersonal skills such as these are known to be significant for ensuring an effective therapeutic relationship (Barker et al. 2014; Hsiao et al. 2015). Nevertheless, it has been shown that individuals are more likely to be responsive and accepting of support from others whom they can identify with (Cruwys et al. 2014) and it is this sense of an affinity to another that fosters the true feelings of trust (Gilchrist et al. 2010). Therefore, nurturing a therapeutic partnership that fosters co-operation (NHSE 2013; Dalum et al. 2015) could offer a more efficacious intervention, as the mutual commitment to the client’s wellbeing and shared sense of endeavour in achieving it, could provide the solidarity that connects them both together (Cruwys et al. 2014). In this study, participants reported that they were included in decision making about their therapy and were assisted by the WBWs to become more resourceful and confident so that they could contribute to their own management. Supporting clients to develop skills, such as these, could be key to promoting a more collaborative relationship (Crepaz-Keay 2010). Further research needs to be undertaken to investigate partnerships working and how this can be optimised in the community setting.

The WBWs were seen by some to offer a flexible service, available when support was required with opportunities for participants to re-attend if there was any future decline in their condition. It is common for people with mental ill health to have relapses throughout their life (Lelliott et al. 2008) therefore, providing an adaptable service with re-attendance at times of most need, will help to prevent further deterioration in symptoms, and provide the chance to nurture individuals back to a point where they can continue to live a more autonomous life. (Coote and MacLeod 2012; Mind 2014). However, offering continued support for all is potentially unsustainable without additional means to provide this, particularly as it is predicted there will be 2 million more people in England with mental ill health by 2030 (MHPG 2014). Certainly, in this study, not all participants perceived flexibility in number of appointments offered and not all received timely access to the WBWs. Indeed, 36% of participants were still waiting for their first appointment by 6 weeks thus, failing to realise the Government’s target for early access (NHSE 2015). Considering the use of other methods including, online resources and wellbeing apps (van’t Hof et al. 2009; Price et al. 2014; Albert et al. 2015), could offer additional means to support individuals in the community to live a more independent life and also, free more time for the WBWs to provide prompt access to their service when most needed. Furthermore, presenting alternative ways for ongoing support could potentially be more motivating to a greater number of people, as it provides more chances for individuals to engage in ways that are perceived as more appealing for them (van’t Hof et al. 2009, Tse et al. 2015). Further research is needed to investigate the efficacy of providing such supplementary resources.

By developing more understanding of their condition and being validated by the WBWs, participants were helped to gain a new perception of their situation and build more positive relationships with others. Reconceptualising their life in this way and developing the confidence to open up and be more communicative also, helped them to reconnect not only with themselves but also, with family and friends. Having healthy relationships with others has been found to foster self-esteem (Connell et al. 2012; Jetten et al. 2015) and lead to improved wellbeing (Cruwys et al. 2014; Greenaway et al. 2015) and gaining family support has been found to be particularly, beneficial to an individual’s emotional health (Sani et al. 2015; Miller et al. 2015). Developing positive social networks in this way and the opportunities for emotional assistance that this affords, could provide an additional resource to support people with mental ill health to self-manage in the community (Haslam et al. 2016; Miller et al. 2015).

## Limitations

There are limitations to this study. The findings of this research are from only one community service and therefore, cannot be deemed transferable to other settings. However, this study offers insight into a practical model of mental health care and the situations described may be seen by the reader to be reflective of people living in similar circumstances.

Although, a number of methods were undertaken to recruit for this study, only 50 individuals chose to take part. Therefore, it is likely that this lacks the views of the passive or disinterested (deWinter et al. 2005) thus, reducing the credibility of the findings and the power of the statistical tests. However, participants did represent a varied cross-section of characteristics including genders, age ranges, employment status and ethnic minority groups. Including open-ended questions provided the opportunity to gain more depth of understanding of the participants’ situation however, it might have dissuaded the less articulate from taking part. Nevertheless, individuals were offered a variety of ways to provide information including, telephone or face to face-to-face interview. Requesting information from individuals who had previously accessed the service relied on participants’ recall, which could affect the authenticity of the findings therefore, more research using a prospective methodology could provide further insights.

## Conclusion

The findings from this study have highlighted the value of an outreach WBS for improving the mental wellbeing of people with psychological distress. The WBWs facilitated participants to become more self-determining by cultivating their resourcefulness to self-manage in a safe and supportive environment. Assisting participants to develop relationships that were more positive helped them to build their social connections thus, offering an additional source of support to self-manage in the community. Nurturing a collaborative partnership by encouraging participants to engage in decision making about their management and instilling within the participants a sense of shared endeavour on their journey to wellbeing, could be key to optimising further, the intervention.

Situated within different settings within the community, the WBS provides a practical example of an adaptable mental health service that can support and promote its citizens to become more resilient and lead a more fulfilling and independent life in the community. However, to ensure its sustainability, the adoption of additional means, including the use of technological resources, to assist the WBWs to continue to support the wellbeing of individuals in the community, needs to be explored. Although this study has demonstrated some encouraging findings, more research with a greater range of participants will provide further insight in to the potential of this service as a model for improving the mental health of individuals in the community.
